# Novel recruitment approaches and operational results for a statewide population Cohort for cancer research: The Healthy Oregon Project

**DOI:** 10.1017/cts.2024.9

**Published:** 2024-01-19

**Authors:** Zhenzhen Zhang, Autumn Shafer, Katie Johnson-Camacho, Andrew Adey, Pavana Anur, Kim A. Brown, Casey Conrad, Rachel Crist, Paige E. Farris, Christina A. Harrington, Lisa K. Marriott, Asia Mitchell, Brian O’Roak, Vanessa Serrato, C. Sue Richards, Paul T. Spellman, Jackilen Shannon

**Affiliations:** 1 Division of Oncological Sciences, Knight Cancer Institute, Oregon Health & Science University, Portland, OR, USA; 2 Department of Medicine, University of California Los Angeles, Los Angeles, CA, USA; 3 Department of Human Genetics, University of California Los Angeles, Los Angeles, CA, USA; 4 Knight Cancer Institute, Oregon Health & Science University, Portland, OR, USA; 5 School of Journalism and Communication, University of Oregon, Eugene, OR, USA; 6 Cancer Early Detection Advanced Research Center, Oregon Health and Science University, Portland, OR, USA; 7 Department of Molecular and Medical Genetics, Oregon Health and Science University, Portland, OR, USA; 8 Knight Cardiovascular Institute, Oregon Health and Science University, Portland, OR, USA; 9 Oregon Clinical and Translational Research Institute, Oregon Health & Science University, Portland, OR, USA; 10 OHSU-PSU School of Public Health, Oregon Health & Science University, Portland, OR, USA; 11 Gene Profiling Shared Resource, Oregon Health & Science University, Portland, OR, USA; 12 Knight Diagnostic Laboratories, Oregon Health & Science University, Portland, OR, USA

**Keywords:** Cohort, population, HOP, genetics, app

## Abstract

**Background:**

Cancer health research relies on large-scale cohorts to derive generalizable results for different populations. While traditional epidemiological cohorts often use costly random sampling or self-motivated, preselected groups, a shift toward health system-based cohorts has emerged. However, such cohorts depend on participants remaining within a single system. Recent consumer engagement models using smartphone-based communication, driving projects, and social media have begun to upend these paradigms.

**Methods:**

We initiated the Healthy Oregon Project (HOP) to support basic and clinical cancer research. HOP study employs a novel, cost-effective remote recruitment approach to effectively establish a large-scale cohort for population-based studies. The recruitment leverages the unique email account, the HOP website, and social media platforms to direct smartphone users to the study app, which facilitates saliva sample collection and survey administration. Monthly newsletters further facilitate engagement and outreach to broader communities.

**Results:**

By the end of 2022, the HOP has enrolled approximately 35,000 participants aged 18–100 years (median = 44.2 years), comprising more than 1% of the Oregon adult population. Among those who have app access, ∼87% provided consent to genetic screening. The HOP monthly email newsletters have an average open rate of 38%. Efforts continue to be made to improve survey response rates.

**Conclusion:**

This study underscores the efficacy of remote recruitment approaches in establishing large-scale cohorts for population-based cancer studies. The implementation of the study facilitates the collection of extensive survey and biological data into a repository that can be broadly shared and supports collaborative clinical and translational research.

## Introduction

Historically, the development of large research cohorts has been limited by the enormous costs of recruitment and retention [1,2]. Additionally, limited bandwidth for interactions and potential loss of participant interest have resulted in reduced follow-up rates, as participants can become overburdened [3,4]. To address these challenges, the prominent epidemiological cohorts of the late 20^th^ century, such as the Framingham Heart Study [5], Physicians’ Health Study [6–9], Nurses’ Health Study [10], and more recent cohort studies, have relied on highly selected populations to enhance compliance and engagement. Of concern, these populations may exhibit behaviors and exposures that are substantively different from the overall population.

In recent years, there has been a transition towards health system-based research and clinical trial cohorts. Nevertheless, these systems usually require participants to be in some sense captive, making it challenging to follow them when they move out of the network, which leads to potential biases in participant retention. Furthermore, these systems are often decoupled from the academic research networks. Fortunately, recent advancement in consumer engagement models utilizing smartphone-based communication, driving projects, and social media have begun to upend these paradigms [11–13].

We sought to develop a research cohort by leveraging smartphones for participant engagement coupled with a specific participant engagement strategy based on inherited cancer DNA screening. We report a highly cost-effective strategy that enables the rapid building of a research cohort, adding 1,500 new participants per month, and reaching more than 1% of the adult population of Oregon in less than two years. Unlike traditional recruitment methods for cohort studies that relied on mass invitation mailings or digital dialing, which are expensive and time-consuming with low response and follow-up rates, our innovative approach overcomes recruitment limitations, making it an ideal fit for translational science.

The research findings from the cohort we are building have the potential to yield valuable research resources for clinical and translational investigations involving diverse patient populations. The goal is to improve participant outcomes by optimizing treatment approaches and enabling accurate outcome predictions, thus providing direct translational insights. Ultimately, our project aims to advance the field of clinical and translational research, fostering a deeper understanding of various health conditions and driving progress toward personalized and effective interventions.

## Innovative population-based cohort-building approaches are needed history of large Cohorts

Large-scale population cohorts established in the second half of the 20^th^ century have facilitated a broad understanding of the major risk factors for diseases, including cancer. In the USA, where no national healthcare system exists, these studies utilized central control and relied on preexisting groups to identify their participants (*e.g.* Framingham Heart Study [5], Nurses’ Health Study [10], and California Teachers Study [14,15]). This approach clearly facilitates communication, engagement, trust, and ultimately compliance with study goals; however, the use of preexisting groups introduces biases that do not reflect the overall population.

## History of electronic medical record (EMR) data repositories

The movement to electronic medical records (EMRs) for billing and treatment histories has allowed the creation of systematically collected data as part of medical care and has facilitated the broad usage of medical records in research. However, EMR-based research systems are not designed for research, and the limitations of engaging with patients have been well-documented [16]. Only the largest health systems can effectively utilize these systems in building research cohorts (e.g. Kaiser Permanente [17], Geisinger Health [18], and the Veterans Administration [19]). Again, these systems are limited by the biases of the individuals covered in these plans as well as the treatment schedules available in these systems.

## New approaches

More recently, projects from the National Institute of Health (NIH)s All of Us Program [11], and two major companies, Ancestry and 23andMe [20], have engaged directly with participants by offering something of value in exchange for participant enrollment. Epidemiological research is becoming a reciprocal process in which participants benefit more directly from studies. These approaches utilize newer technologies, particularly smartphone apps, to decrease barriers to engagement and communication. These approaches actively encourage direct participation in research by community members. We sought to capitalize on these trends to engage with the population of the state of Oregon, enabling broad research engagement at a low cost by utilizing a specific driving project, genetic health screening.

## Materials and methods

### Study overview

The Healthy Oregon Project (HOP) study was approved by the Oregon Health & Science University (OHSU) Institutional Review Board (IRB #STUDY00018473). Data from HOP surveys and DNA samples are securely stored by OHSU in a research repository accessible to scientists approved by the HOP and OHSUs research ethics board who are working on cancer early detection research and effective treatments for inherited diseases.

### Recruitment strategy

The driving recruitment model for this study was to market the study across the state to drive individuals to download the study app which allowed for eligibility assessment, consent, and surveys. The inclusion criteria encompass all adults in Oregon, which includes cancer survivors. Participants consenting to genetic health screening either visited a location in the community for a collection kit returned on-site, or utilized a mail-in option, where the participant requested a kit within the app to be sent to their Oregon address.

### Internet/media

The recruitment and participant support for the study were facilitated through the unique email account healthyoregonproject@ohsu.edu and the webpage https://healthyoregonproject.com/. We utilized a study landing page on the HOP website that directed smartphone users to the appropriate app store for their devices. HOP uses a variety of social media platforms, including Facebook and Twitter, to engage with participants and direct them to the appropriate app store.

The HOP project also delivers monthly newsletters by email to participants to share with friends, family, or community groups to help spread the word about the HOP. Participants were encouraged to share the study broadly by sharing a post on their social media, sending the HOP website (HealthyOregonProject.com) to their go-to group chat, or telling their families about the results they received.

### Healthy Oregon Project (HOP) app

The HOP app can be downloaded on participants’ smartphones. After consent, the participants can engage with the app through two components of the study: (a) one is a series of surveys and (b) the other is saliva sample collection. *(a) HOP Surveys:* The primary data collection administered through the HOP app includes questions on (1) behaviors: examining the relationship between chronic disease risk and behaviors like smoking cigarettes and drinking alcohol; (2) cancer history: helping researchers to understand past cancer diagnosis and known family history; (3) colorectal history: personal and family history related to colorectal cancer and disease; (4) lifestyle: everyday lifestyle to help researchers understand the participants and the health impacts; (5) stress: experiences with stress over the last month before the survey; and (6) COVID-19: understanding how the pandemic has affected cancer risk, prevention, and screening behaviors. Later, (7) a measure of impulsivity and (8) a test of reaction time were added to provide tips for healthy sleep, decision-making, and lifestyle choices. With the ongoing study, more questions will be supplemented through the HOP app, and participants will receive notifications through email. We aimed to obtain detailed data regarding risk factors impacting early cancer detection to enhance the breadth of the data with additional targeted support. Our ultimate goal was to identify strategies for early detection of cancer. *(b) Biological specimen collections for inherited genetic health screening:* Following initial consent, the participants may click “Enroll: Genetic Screening” in the app’s Activities tab to complete additional consent for genetic testing. Following consent, participants can request saliva sample collection kits through the “Activity: Request a DNA Kit” action on the HOP app. The study implemented a mail option, where participants requested a DNA self-collection kit to be delivered to an Oregon address and then used the provided return mailing envelope to send to the screening lab. The participants were instructed that within 24 h of collecting the samples, they would drop-off their sealed envelopes through home mailboxes, at local post offices, or in a U.S. Post Office blue collection box. Participants were informed that results from DNA health screening would be returned at no cost within six months of receipt.

### Genes screened and reporting

DNA samples were sequenced at OHSU Knight Diagnostic Laboratories, a Clinical Laboratory Improvement Amendments lab at OHSU. Processes for genetic screening were covered in O’Brien *et al*. [21] According to the IRB-approved protocol, only pathogenic and likely pathogenic variants for which National Comprehensive Cancer Network (NCCN) guidelines exist are returned to the participants. All other participants received a negative report explaining that no variants that affected their care were detected. All participants with a positive test received genetic counseling at no cost from the OHSU genetic counseling staff.

## Results

**Figure 1.**
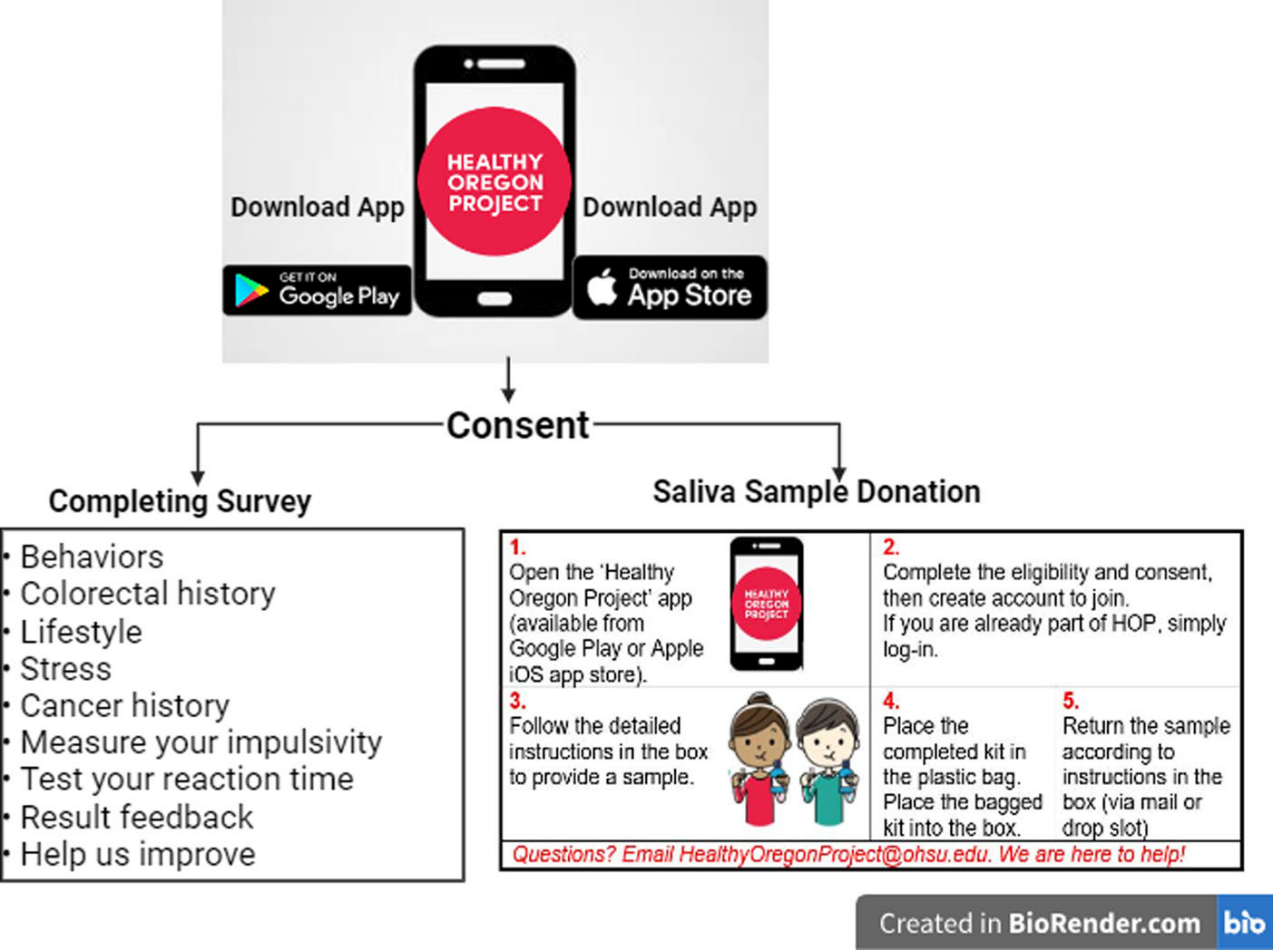
Overview of the Healthy Oregon Project study process.

The HOP is an IRB-approved research protocol (IRB #STUDY00018473) that addresses the challenge of engaging with the catchment area of the Knight Cancer Institute (KCI), an NCI Comprehensive Cancer Center that serves the state of Oregon. The KCI leadership contracted with a Health Insurance Portability and Accountability Act-compliant smartphone app vendor to deploy a consenting and enrollment system available on iOS and Android devices through their respective app stores (Fig. 1). Consents were developed in close collaboration with IRB staff and included general research consent as well as additional consent for secondary studies, including a driving project to identify community members with inherited diseases who have established interventions that significantly alter patient health.

**Figure 2.**
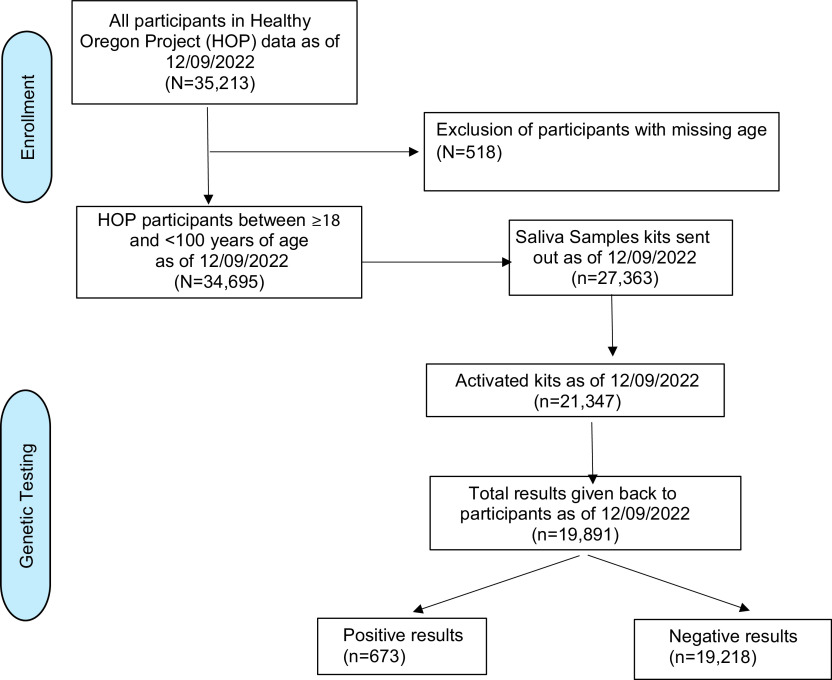
Study population flowchart.

Primary genetic health screening was performed to detect genetic variants that increased the risk of cancer, particularly hereditary breast and ovarian cancer syndrome, Lynch syndrome, and less frequent syndromes such as Cowden’s syndrome and Li Fraumeni Syndrome. Prior to the launch of genetic health screening, we performed a community readiness assessment including focus groups [22]. As genetic health screening is offered to those who do not meet the NCCN genetic testing guidelines, only variants that are deemed pathogenic or likely pathogenic are identified by the testing lab and returned to the participants (see Methods). The full list of genes screened is shown in Table 1 and additional information on genetic health screening has recently been published [21]. HOP was designed to utilize direct participant engagement strategies, including direct mail, earned media, direct engagement, and social media. Enrollment in HOP occurred in two phases: a pre-COVID phase of in-person events and vending machine kit dispensing, and a post-COVID phase where mail-based recruitment became the standard. The in-person phase employed both small-scale human interactions (e.g., health fairs), engagement with large employers, and the use of kit dispensing machines situated at specific locations. Figure 2 provides an overview of the effectiveness of recruitment and total numbers. In total, 4,810 participants were recruited between 12/01/2018 and 10/16/2020 using in-person approaches. The shift to mail-based enrollment on Oct 17, 2020, resulted in the successful recruitment of a total of 34,695 participants (as of December 09, 2022), with valid age information. Individuals who downloaded the app but did not provide consent were not considered study participants.

**Table 1.** Table of genes screened for deleterious variants

*APC*	*BRCA2*	*CHEK2*	*MSH6*	*POLD1*	*RET*	*TSC2*
*ATM*	*BRIP1*	*MEN1*	*MUTYH*	*PTEN*	*SMAD4*	*LDLR*
*BAP1*	*CDH1*	*MITF*	*NBN*	*RAD51C*	*STK11*	
*BMPR1A*	*CDK4*	*MLH1*	*PALB2*	*RAD51D*	*TP53*	
*BRCA1*	*CDKN2A*	*MSH2*	*PMS2*	*RB1*	*TSC1*	

**Figure 3.**
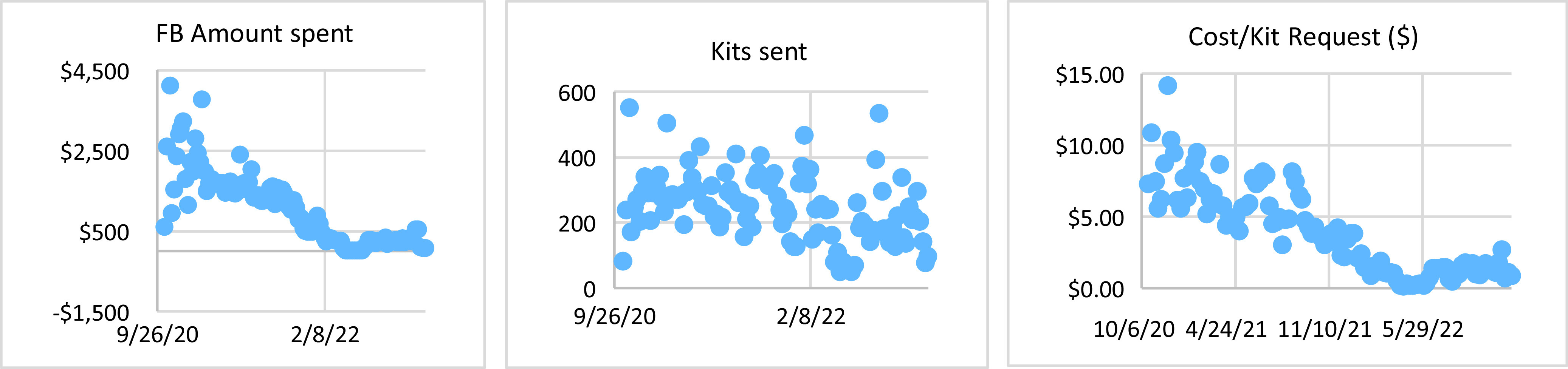
Cost per kit request was used as a surrogate of overall effectiveness. Weekly spend (***a***) was adjusted regularly to maintain a running average of 300 kit requests per week (***b***). Spending per week ranged from a high of $4,123 (week of 11/4/2020) to a low of $14 (week of 5/16/22).

After switching to mail-based recruitment, we were able to directly measure the efficacy of social media, earned media, and digital communication with participants on engagement. One key parameter of such an engagement is the cost per enrolled participant. We found that after a relatively short training period of approximately eight months, social media systems moved from being very effective (approximately $10/kit request) to extremely effective (approximately $1.5/kit request), as shown in Figure 3. The saliva kit costs, including labor, around $5 per participant. When two-way postage and a 70% return rate are taken into consideration, the total cost is approximately $10/participant. The cost of genetic testing per participant was around $75. The genetic counseling was provided at a cost of $500 per positive result. Given the positivity rate of approximately 5%, the average cost per participant for genetic counseling was around $25.

Due to laboratory constraints that limited throughput, we dramatically curtailed advertising in April and May of 2022; the average weekly spend was $22, compared to $283 in February and March, and $224 in June and July. Enrollment decreased to 80 participants/week during the lowered spending versus 238 and 251 per week in the two months prior and following, respectively. Nonetheless, baseline enrollment was presumably maintained through social networking effects. The HOP monthly email newsletters have an average open rate of 38% with a range from 27% to 56% from the study start date to Dec. 2022. This is much higher than the industry average and the survey reminder within the monthly newsletter typically receives 17.1% of the clicks each month, indicating a substantial level of reader interaction.

Efforts were made and continue to be made to improve survey response rates, such as email reminders about the surveys in general and specific surveys. Each month, one survey is featured in the newsletter to highlight its importance along with a link to the app to complete it. Social media campaigns with both organic posts on HOP’s social pages and also ads targeted towards people who have clicked an app download ad or followed HOP include appeals to complete the surveys. Continuous efficiency testing occurred for the HOP ads and organic social media posts. Meta’s Ads Experiments feature allows for A/B testing of ads to compare click rates, reach, app downloads, and website views of ads that are randomly assigned to groups within a selected target audience. HOP tested message attributes, such as visuals, wording, ad format, and message frames to improve ad efficiency. An example of such testing was a comparison of a local cue in the ad that either stated, “Oregon residents are invited to join HOP” or “Lane County residents” (tailored to each county the ad was shown in), which found that the localized county ads were 43% cheaper per website view than the “Oregon” ads. Another example was a comparison of a theme focused on the personal benefit of participating (knowing your cancer risk) vs. an ad with identical esthetics, but the text was themed around the altruistic benefit (contributing to cancer research). The personal benefit-themed ad was 37% cheaper per website view than the altruistic benefit ad.

We further measured the effectiveness of additional engagement through newsletters in our population, where the cost per participant in the weeks following an engagement email to those already identified by low-cost lead generation campaigns led to a median 19% decrease in costs for the week after an email newsletter compared to the week before the newsletter (Table 2). No effects were observed between the week after the newsletter and the subsequent two weeks.

**Table 2.** Improvement in cost-effectiveness associated with outreach

	Cost per kit request		
Date	Week before newsletter	Week after newsletter	Decrease in cost (%)
10/26/20	10.87	7.48	0.31
11/30/20	14.15	10.36	0.27
12/21/20	6.17	5.61	0.09
1/26/21	8.85	9.51	−0.07
2/22/21	5.21	6.22	−0.20
3/22/21	8.64	5.78	0.33
4/26/21	4.97	4.01	0.19
5/24/21	5.91	7.68	−0.30
6/28/21	7.93	5.78	0.27
7/26/21	4.98	3.05	0.39
8/30/21	7.46	6.48	0.13
9/27/21	4.29	3.81	0.11
10/25/21	3.53	3.03	0.14
11/29/21	4.25	2.32	0.45
1/31/22	1.46	0.90	0.38
2/28/22	1.89	1.13	0.41
3/28/22	1.07	0.52	0.52
6/27/22	1.41	1.39	0.02
7/25/22	0.66	0.48	0.27
8/29/22	1.81	1.53	0.16
9/28/22	0.96	1.48	−0.54
10/25/22	1.34	1.09	0.18
11/28/22	1.11	0.86	0.23
Median			0.19

The structure of smartphone engagement encourages short and frequent interaction. This approach is not highly conducive to data collection through long- or multi-part surveys, as is often observed in traditional cohort studies. However, by splitting surveys into multiple short and straightforward engagements, participants can complete numerous research surveys when convenient, and it is possible to control the order of survey completion by making the survey available only to participants who have completed other surveys within the app. When possible, the research surveys were designed to be completed in five minutes, maximizing convenience to the participant and reducing the burden on any one sitting. In total, seven surveys were initially made available, and depending on the survey, 27%–72% of the participants completed each survey. A full breakdown of all participants and the number of completed surveys is presented in Table 3.

**Table 3.** Completion rates of study surveys by survey items

Survey items^1^	*N* (Percent)
Total N accessing the survey app	35,973
HOP general consent	31,147 (86.6%)
Behaviors	25,266 (70.2%)
Cancer history	22,621 (62.9%)
Colorectal history	19,746 (54.9%)
Lifestyle	20,099 (55.9%)
Perceived Stress	18,358 (52.1%)
COVID and cancer^2^	1900 (5.3%)
Impulsivity^3^	11,665 (33.1%)
Test of reaction time (vigilance)^3^	9945 (28.2%)
Results feedback	9200 (25.6%)

1 The survey completion rates results were mainly based on data from the start of the study 12/01/2018 to 01/20/2023. The perceived stress, impulsivity and vigilance results were based on data from the study start date to 12/09/2022 (total *N* = 35,213). All surveys are available to all participants via the app, including those added at different dates. However, participants are only required to complete the initial demographics and consents surveys.

2 The COVID and cancer survey was later added into the HOP app as part of an NIH supplement grant, which was mostly active during Aug. 2020–Jan. 2021.

3 Impulsivity and vigilance surveys were later added into the HOP app on 08/03/2021.

### Participant characteristics

The demographics of the in-person cohort and mail-based cohort recruited from the start until December 9, 2022, are presented in Tables 4 and 5. Consistent with the convenience sampling approach, the study population was not randomized. Our analytical cohort included 34,695 participants aged 18–100 years (media*n* = 44.2 years). Distributions for demographic and behavioral factors of HOP Participants are: 72.3% lived in urban areas, 75.3% were females, 83.0% were white, 36.7% were obese, and 28.7% were overweight. Regarding behavior-related factors, 39.1% had smoked at least 100 cigarettes in their lives, and 92.7% had consumed at least 30 alcoholic beverages in their lives. Most participants had inadequate fruit and vegetable intake, with 70.3% having ≤ 1 cup/day of fruit intake and 59.1% having ≤ 1 cup/day of vegetable intake. The majority of participants did not report regular exercise or exercised for less than 1 h/day (56.2%) during weekdays. Most participants reported more than 8 h/day sleep (54.5% during weekdays and 56.1% during weekends).

**Table 4.** Demographic characteristics of Healthy Oregon Project (HOP) participants

Participant characteristics	HOP participants *n* (%)	Oregon population^2^
	Total *N* = 34,695	3,402,545
**Rural/Urban Status** ^3^		
Urban	24,079 (72.3%)	65%
Rural	9,214 (27.6%)	33%
Unknown/other states	474	N/A
Undefined	928	N/A
**Age Group^1^ (media*n* = 44.2; mean *SD* = 46.8, 14.4)**		
18– < 35	7,906 (22.8%)	28%
35– < 50	13,910 (40.1%)	25%
50– < 65	8,025 (23.1%)	23%
≥65	4,854(14.0%)	24%
**Gender**		
Female	26,111 (75.3%)	50.1%
Male	7,945 (22.9%)	40.9%
Unknown or undefined	639 (1.8%)	
**Race/Ethnicity (*n* = 35,213)**		
White	28,793 (83.0%)	73.5%
Black or African American	411 (1.2%)	2.3%
American Indian or Alaska Native	469 (1.4%)	1.9%
Asian	1,281 (3.7%)	5.1%
Native Hawaiian Pacific Islander	180 (0.5%)	0.5%
Middle Eastern/North African	67 (0.2%)	N/A
Hispanic	2,164 (6.2%)	14.4%
Others	290 (0.8%)	1%
Unknown or undefined	1,108 (3.2%)	N/A

1 We included consented participants with valid age information into the descriptive analysis.

2 Oregon population data were accessed through US Census Bureau, QuickFacts: Oregon. https://www.census.gov/quickfacts/OR. The total N shows OR adult population ≥ 18 years old. More refined age distribution data were obtained from https://censusreporter.org/profiles/04000US41-oregon/ and https://www.statista.com/statistics/1022743/oregon-population-share-age-group/.

3 Urban/rural status was accessed through Oregon Office of Rural Health. https://www.ohsu.edu/oregon-office-of-rural-health/about-rural-and-frontier-data.

**Table 5.** Lifestyle characteristics of healthy oregon project (HOP) participants

Participant characteristics	HOP participants *n* (%)
BMI (*n* = 20,234)	
Underweight	253 (1.3%)
Healthy weight	6699 (33.1%)
Overweight	5805 (28.7%)
Obese	7477 (36.9%)
**Cigarette smoking (*n* = 24,701)^1^**	
Yes	9665 (39.1%)
No	15,046 (60.9%)
**Alcohol intake (*n* = 24,711)^2^**	
Yes	22,918 (92.7%)
No	1793 (7.3%)
**Current alcohol drinker (*n* = 22,918)^3^**	
Yes	16,579 (72.3%)
No	6339 (27.7%)
**Ever Had Cancer (*n* = 20,233)^4^**	
Yes	3177 (15.7%)
No	17,056 (84.3%)
**Vegetables intake (cups/day) (*n* = 20,233)^5^**	
0	510 (2.5%)
1	11,442 (56.6%)
2	5305 (26.2%)
3	2001 (9.9%)
4	975 (4.8%)
**Fruits intake (cups/day) (*n* = 20,233)^6^**	
0	964 (4.8%)
1	13,242 (65.5%)
2	4370 (21.6%)
3	1282 (6.3%)
4	375 (1.9%)
**Exercise weekday (hours/day) (*n* = 20,234)^7^**	
None	1604 (7.9%)
>0 – ≤ 1	9776 (48.3%)
2	6972 (34.5%)
≥3	1882 (9.3%)
**Exercise weekend (hours/day) (*n* = 20,234)^8^**	
None	1980 (9.8%)
>0 – ≤ 1	7735 (38.2%)
2	8330 (41.2%)
≥3	2189 (10.8%)
**Sleep weekday (hours/day) (*n* = 20,234)^9^**	
<8	9209 (45.5%)
≥8	11,025 (54.5%)
**Sleep weekend (hours/day) (*n* = 20,234)^10^**	
<8	8888 (43.9%)
≥8	11,346 (56.1%)
**General health (*n* = 20,234)^11^**	
Excellent	1504 (7.4%)
Very good	6004 (29.7%)
Good	8105 (40.1%)
Fair	3818 (18.9%)
Poor	803 (4.0%)

Original survey questions (The analyses were based on data received from beginning of the study to 12/09/2022).

1 Smoking: Have you smoked at least 100 cigarettes in your ENTIRE life?.

2 Alcohol: Have you had at least 30 alcoholic beverages in your ENTIRE life?.

3 Current alcohol drink: Do you CURRENTLY drink alcoholic beverages (beer, wine, liquor, cocktails, coolers)?.

4 Ever had cancer: Has a physician ever told you that you have CANCER (not including basal or squamous cell skin cancer or cervical dysplasia)?.

5 Vegetables: About how many cups of VEGETABLES (other than iceberg lettuce and potatoes) do you eat PER DAY?.

6 Fruits: About how many cups of FRUIT (including 100% pure fruit juice) do you eat or drink PER DAY?.

7 Exercise weekday: Exercise/physical activity (hours per day WEEKDAYS M-F).

8 Exercise weekend: Exercise/physical activity (hours per day WEEKENDS Sat & Sun).

9 Sleep weekday: Sleeping (hours per day on WEEKDAYS M-F).

10 Sleep weekend: Sleeping (hours per day WEEKENDS Sat & Sun).

11 General health: In general, would you say your physical health is.

### The expanding research opportunities and impact of the HOP study

One key feature of HOP is that it can easily provide additional research opportunities. When HOP participants initially consented to the study, they were informed about the potential for receiving invitations to participate in additional studies.

Subsequent studies launched within HOP included studies that have included the collection of additional data and specimens, for example, collection of fecal samples and wristband exposure data to understand the association between the microbiome, environmental chemical exposure, and measures of impulsivity and vigilance (National Institute of Environmental Health Sciences of the National Institutes of Health grant number: P30ES030287); targeted recruitment for partner institution studies, such as a prospective observational cohort study to assess SARS-CoV-2 vaccine effectiveness in children and adults (CDC Contract number:75D30121C12297), and older adult cancer survivors to engage in home monitoring (supported by the Knight Cancer Institute’s Cancer Center grant number: P30CA69533).

Of great importance, after the launch of HOP, the NCI Moonshot program funded U01CA232819 to evaluate the cost-effectiveness of broad HBOC and Lynch syndrome screening in the HOP population (NCT04494945). We previously published [21] a breakdown of genetic testing results from the first 13,774 results returned to the HOP participants. Briefly, just over 5% percent of HOP genetic health screenings resulted in a test that required confirmation and genetic counseling. Complete collection of data for NCT04494945 is expected in the summer of 2025.

In our ancillary studies derived from HOP, the response rate is contingent on the particular study. For instance, the microbiome study boasts a recruitment rate of 90% due to targeted invitations sent to a specific group. Conversely, when we have reached out to HOP participants via a single email to offer opportunities to participate in external studies, the email open rate has ranged between 40–55% with approximately 12–15% of participants clicking through to the external study site, and either completing surveys or completing eligibility to enroll in the study.

## Discussion

The HOP study applied novel recruitment approaches to rapidly engage with a statewide population. Prior to the onset of COVID-19, our primary recruitment methods were in-person community events and the installation of vending machines for pick-up/drop-off genetic screening kits. With the onset of COVID-19, we shifted our recruitment model to a remote approach by marketing the study across the state through multiple social media campaigns, both reaching the broad general population and more specifically targeted efforts at hard-to-reach under-recruited populations. The HOP’s goal was to contribute to research that is convenient, accessible, and mutually beneficial.

The participant-driven model based on engagement with the questions utilized in the cohort and the prospect of return of information was widely acceptable. Concerns on social media were primarily focused on genetic health screening in HOP and commonly expressed the idea that genetic testing results might impact health or life insurance. Less commonly, social media concerns focused on intrusiveness, concerns about sharing DNA, and privacy rights. Engagement with HOP staff through social media and direct electronic communications pointed potential participants to web resources, including the HOP website (www.healthyoregonproject.com), which informed potential participants on security, confidentiality, and federal or state regulations that prevent discrimination in health care based on inherited genetic differences.

However, two key challenges remain to be addressed. First, we do not yet have firm measures of how well we will continue to engage with participants or how long the engagement will last. Long-term engagement is necessary to facilitate the secondary research projects that HOP is designed to support. Second, HOP represents a single small catchment area that is demographically quite uniform, as Oregon is among the oldest and least diverse states in the US. Broad centralized national cohorts such as Veterans Affairs’ Million Veteran Program and NIH’s All of Us are certainly other solutions to the demographic limitations of the HOP. However, we imagine another scenario in which HOP could integrate with an array of similar projects across the US to facilitate expansion or validation cohorts for questions arising from those diverse cohorts. Cohort integration would diversify the population base and democratize access to cohorts for researchers. Finally, these cohorts could create an expanded array of driving projects, and the community of cohorts could evaluate how best to bring the community into research. A network of cohorts utilizing the same platforms and similar consents could engage with 100,000 participants nationwide sharing access to data and research questions broadly.

## Conclusions

The HOP study provides a novel, remote-based, inexpensive recruitment approach to effectively establish a large-scale cohort for population-based studies of cancer. This study provides evidence that remote recruitment approaches can effectively establish large-scale cohorts for population-based cancer studies. The implementation of the study facilitates the collection of extensive survey and biological data into a repository that can be broadly shared and supports the creation of cohorts available for collaborative clinical and translational research.

## Data Availability

Our study has not been published elsewhere nor has it been submitted simultaneously for publication elsewhere.
